# Prevalence and Impact of Period and Pelvic Pain in Australian Adolescents: The PPEP Talk Schools Program

**DOI:** 10.1111/ajo.70015

**Published:** 2025-03-14

**Authors:** Kate I. Tomsett, Amelia K. Mardon, Olivia W. Gao, Annabelle K. Simpson, Bridie C. Squire, Indigo G. Warner, Susan F. Evans

**Affiliations:** ^1^ Pelvic Pain Foundation of Australia Adelaide South Australia Australia; ^2^ Reproductive Health Western Sydney University Sydney New South Wales Australia; ^3^ Faculty of Health and Medical Sciences University of Adelaide Adelaide South Australia Australia; ^4^ University of Adelaide Adelaide South Australia Australia

**Keywords:** adolescent, education, endometriosis, pelvic pain

## Abstract

**Background:**

In 2018 the Australian Government launched the world's first National Action Plan for Endometriosis (NAPE). Of its three priorities ‘Priority 1’ was ‘Education and Awareness’. In response, the Pelvic Pain Foundation of Australia was funded to deliver their Periods, Pain and Endometriosis Program (PPEP) Talk to a proportion of Australian schools. Since then, PPEP Talk has been delivered to over 110,000 students.

**Aims:**

This retrospective cross‐sectional study investigated students assigned female at birth (AFAB) and the prevalence and impact of period and pelvic pain, interaction with health care services and knowledge of endometriosis.

**Materials and Methods:**

Multiple choice, pre and post PPEP Talk, paper survey responses between July 2022 and June 2023 were collected from 13,078 students AFAB.

**Results:**

52.6% of students reported regular severe period pain. 22.9% of students reported regularly missing school or work with their period. 21.5% of students had presented to a health professional for pain, and 5.7% had presented to an Emergency Department. 5.2% of students reported pelvic pain for more than 10 days per month. The prevalence and impact of period and pelvic pain varied across demographic variables. The proportion of students who knew what endometriosis was rose from 47.8% to 95.5% after the program.

**Conclusions:**

The NAPE's objective to enhance education and awareness of endometriosis and period/pelvic pain was met. 100% of schools who received PPEP Talk want it to return. Wide discrepancies in the prevalence of pain within different demographics were identified, providing previously unknown data to improve and direct services.

## 1 Introduction

Endometriosis can be defined as an inflammatory condition whereby endometrial‐like tissue grows outside of the uterus [1]. It affects approximately one in seven Australian adult women [2]. Endometriosis and persistent pelvic pain (PPP) place a considerable burden on adolescents [3]. However, there is a lack of large‐scale studies investigating the prevalence and impact of period and pelvic pain among Australian students. In 2018 the Australian Government polled stakeholders throughout the health system including medical researchers, health professionals and consumer groups on their priorities for inclusion in Australia's National Action Plan for Endometriosis (NAPE) [4]. The Pelvic Pain Foundation of Australia (PPFA) was funded by the Federal Department of Health and Aged Care to address the schools education component of Priority 1 of the NAPE: ‘Education and Awareness’. With the additional support of state governments, PPEP Talk expanded incrementally, to reach a proportion of schools in, all states by 2022.

Australian adolescents face considerable impacts due to PPP. One cross‐sectional study (*n* = 1051) demonstrated that 93% of Australian adolescents experience pain with menstruation, 21% experience severe period pain, and 26% had not attended school or work because of their period [5]. Teens with higher scores of menstrual pain were found to be more likely to experience higher rates of absenteeism [6]. Further, PPP and endometriosis negatively impact adolescents' mental health, social wellbeing and quality of life [7]. The early recognition of symptoms suggestive of endometriosis or pelvic pain provide an opportunity for early diagnosis and interventions that are critical to improving the wellbeing of adolescents.

Endometriosis and PPP impose a large burden on health and workplace economies. In 2019 the economic burden of endometriosis per annum in Australia was estimated to be between $7.4 billion and $9.7 billion [8]. Lost productivity due to endometriosis accounted for 84% of the $7.4 billion estimate and cost increased with pain severity [2].

### 1.1 The PPEP Talk

Funded through a combination of Federal and individual State Governments (Queensland, Victoria, Tasmania, South Australia and Western Australia), the free, 90‐min, in‐person sessions were accessible to schools nationwide (NW) up to a funded limit for each state. The availability of the program was publicised to schools aiming for a representative mix of varied demographics. The program targeted students in Grade 10 (aged 15–16) and above. It provided evidence‐based and medically accurate information about endometriosis, period and pelvic pain, incorporating modern neuroscience pain concepts. The program helped students understand whether their pain was normal; explained how the wide range of symptoms they may have experienced are linked together; explained what endometriosis is and the treatments available; provided advice on integrating simple self‐management techniques for pain; and how to seek the advice of a health practitioner where pain persists. To allow for sensitivities within different school communities each school was consulted to ensure that PPEP Talk educators were able to provide a tailored approach to individual schools. All clinical educators who delivered the program were tertiary health qualified and received comprehensive on‐going training in endometriosis, pain science, multidisciplinary care, the biopsychosocial approach, presentation skills and the education of teens. The scaffolded learning experience for students comprised a pre‐PPEP Talk video outlining basic anatomy; the interactive in‐person PPEP Talk session; one‐on‐one time with the PPEP Talk clinical educators for personal concerns; and a free online PPEP Talk Next Steps session where students and parents/caregivers can speak with a gynaecologist, and a pelvic physiotherapist. To ensure continuity of care, ‘wrap around’ supports were provided including the provision of resources, education of the school community (nurses, teachers), meetings with local health professionals and additional sessions within local community centres.

To respond to specific community needs and priority groups, modifications of the basic PPEP Talk program were designed. These included PPEP Talk for students assigned male at birth (AMAB), co‐educational sessions with both AFAB and AMAB students, PPEP Talk Sports, PPEP Talk and Yarns (First Nations students), PPEP Talk for Trans and Gender Diverse Teens, PPEP Talk Workplace, PPEP Talk for Health Professionals, PPEP Talk for Community Groups, PPEP Talk for people living with Intellectual and Developmental Disabilities (IDD), people within the juvenile and adult justice system, and culturally and linguistically diverse (CALD) groups. Responses from these programs have been included and will be compared to the mainstream model where further research funding allows.

### 1.2 Aim

The aim of this study was to retrospectively investigate the prevalence of period and pelvic pain symptoms and the frequency of presentation to health professionals among students assigned female at birth (AFAB) who received PPEP Talk from the 1st of July 2022 to the 30th of June 2023.

## 2 Materials and Methods

This study is a retrospective cross‐sectional analysis of paper survey evaluation data collected from 13,078 students before and after receiving a PPEP Talk session between July 1st 2022 and June 30th 2023. Ethics approval for this timeframe was granted by the University of Adelaide Human Research Ethics Committee (HREC) (H‐2023‐292).

### 2.1 Sample

Survey respondents were Australian students AFAB, aged 15–18, who completed a paper‐based survey prior to and post receiving the PPEP Talk in the 22–23 financial year (FY). Data is available from Catholic and Independent schools in every State and Territory, and Government schools in Queensland, Victoria, South Australia and Western Australia.

### 2.2 Nationwide Demographics

**FIGURE 1:**
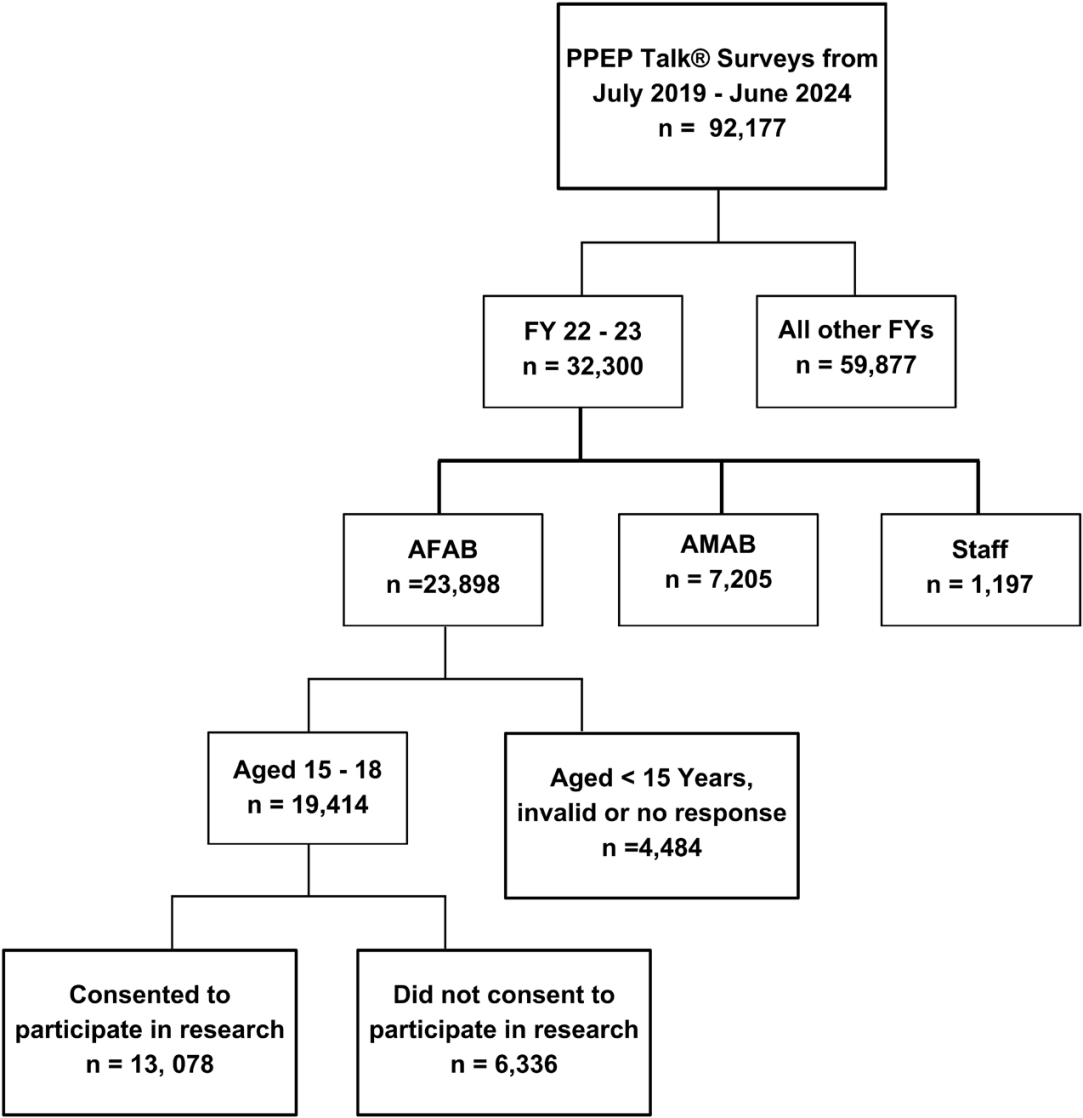
Consort diagram outlining the study inclusion criteria. Total population *n* = 92,177 and sample size *n* = 13,078.

In the 2022–2023 financial year, 23,898 students AFAB received the PPEP Talk and completed the evaluation form. Following the application of age and consent to participate constraints 13,078 students' responses were eligible for inclusion in the study (Figure 1). Over half (54.3%) of the cohort were aged 15 years and 30% resided in Queensland (Table 1).

**TABLE 1:** Demographic data of sample.

Characteristic	*n* (%)
Age	
15	7166 (54.3)
16	4393 (33.3)
17	1356 (10.3)
18 or older	163 (1.2)
Gender	
Female	12482 (97.1)
Male	99 (0.8)
Non‐binary	193 (1.5)
Prefer not to say	85 (0.7)
No response/invalid	219
National identity	
Aboriginal	530 (4.0)
Torres Strait Islander	31 (0.2)
Both	60 (0.5)
Neither	12289 (93.0)
No response/invalid	168
State/territory of residence	
Queensland (QLD)	3962 (30.3)
New South Wales (NSW)	2050 (15.7)
Australian Capital Territory (ACT)	418 (3.2)
Victoria (VIC)	1583 (12.1)
Tasmania (TAS)	256 (2.0)
South Australia (SA)	2602 (19.9)
Western Australia (WA)	2084 (15.9)
Northern Territory (NT)	123 (0.9)

### 2.3 Inclusion Criteria

Complete pre and post survey responses from students who were assigned female at birth, aged 15 years or older, were sufficiently fluent in English to complete the survey, and who provided consent for study participation were included in the research project.

### 2.4 Exclusion Criteria

If post survey completion was not attempted, students were assigned male at birth, or students received the PPEP Talk prior to July 1st 2022 or after June 30th 2023 these responses were not included.

## 3 Procedure

After the first survey in July 2021, survey iterations were trialled to determine the most effective question selection. Survey data for this study was collected from July 1st 2022 to June 30 th 2023 (see Supporting Information for survey).

Clinical educators distributed a physical (paper) copy of the survey form to be completed immediately pre and post PPEP Talk. The anonymous survey collected categorical demographic data and feedback on PPEP Talk. Embedded within the survey for those AFAB was the Period ImPact and Pelvic Pain Assessment (PIPPA) tool to assess the impact of period and pelvic pain [9]. A full assessment of PIPPA outcomes will be reported in a future paper. The five questions analysed in this report are:

Over the last 6 months have you:

1. Had regular severe period pain?

2. Regularly missed school or work because of your period?

3. How many days on average a month would you have pelvic pain or discomfort of any kind? (out of 30 days).

Over your lifetime have you:

4. Seen a health professional (GP, physiotherapist, psychologist, dietician etc.) about period/pelvic pain?

5. Been to an emergency department at a hospital for pelvic pain?

After the PPEP Talk presentation, the physical surveys were scanned and uploaded into papersurvey.io. Papersurvery.io used an optical character recognition (OCR) model to extract and store responses in a database. To ensure accuracy, sensitivity, transparency and to protect against bias, all data was randomly assigned and internally audited quarterly. All blank, invalid and inconsistent responses were accounted for and 5% of the data was randomly selected for verification. According to ethics requirements (see Figure 1) any response from a student who was under the age of 15 and did not consent to the use of their data was removed. Any survey forms returned blank were removed from the sample. In Queensland (makes up 30% of the sample) the physical number of students versus the count of survey forms returned was trialled, and the return rate was greater than 95%. Raw and deidentified data was exported to password protected excel files for cleaning and analysis by an independent group of medical students from the University of Adelaide (OG, AS, BS, IW).

### 3.1 Data Analysis

A retrospective analysis of audit data was performed in Microsoft Excel using descriptive statistics. Data from all students AFAB was anaylsed, from the identified questions. Data was divided into State and Territory demographic variables (metropolitan, non‐metropolitan, Independent, Catholic and Government schools and ICSEA (Index of Community Socio‐educational Advantage)). Null responses and invalid answers were excluded from analysis. Comparisons were made between each group using chi‐squared tests.

## 4 Results

### 4.1 Period and Pelvic Pain Impact by State and Territory

Comparatively Tasmanian students demonstrated the greatest impact of period and pelvic pain with the highest rate of absenteeism (28.6%) and presentation to a health professional (HP) (30.2%) (Table 2). Students from the NT reported the greatest rate of regular severe period pain (60.7%) and students from NSW reported the lowest (45.1%). Rates of presentation to a HP and to ED were lowest in the NT (14.6% and 2.5% respectively) with all other states/territories close to the national average (21.5% and 5.7% respectively).

**TABLE 2:** Impact of period and pelvic pain on Australian adolescents AFAB by State and Territory.

Had regular severe period pain? *n* (%)								
QLD	NSW	ACT	VIC	TAS	SA	WA	NT	Nationwide
2150 (54.75)	913 (45.11)	193 (47.19)	833 (53.57)	141 (55.73)	1372 (53.14)	1125 (54.45)	74 (60.66)	6801 (52.57)
Regularly missed school or work because of your period? *n* (%)								
QLD	NSW	ACT	VIC	TAS	SA	WA	NT	Nationwide
971 (24.69)	348 (17.13)	92 (22.38)	309 (19.83)	73 (28.63)	649 (25.12)	501 (24.24)	22 (17.89)	2965 (22.87)
Seen a health professional about period/pelvic pain? *n* (%)								
QLD	NSW	ACT	VIC	TAS	SA	WA	NT	Nationwide
880 (22.36)	413 (20.34)	87 (21.22)	329 (21.09)	77 (30.20)	564 (21.81)	423 (20.42)	18 (14.63)	2791 (21.52)
Been to an emergency department at a hospital for period/pelvic pain? *n* (%)								
QLD	NSW	ACT	VIC	TAS	SA	WA	NT	Nationwide
226 (5.78)	100 (4.94)	26 (6.31)	74 (4.80)	17 (4.52)	159 (6.17)	131 (6.37)	3 (2.46)	736 (5.71)

### 4.2 Days of Pelvic Pain

**FIGURE 2:**
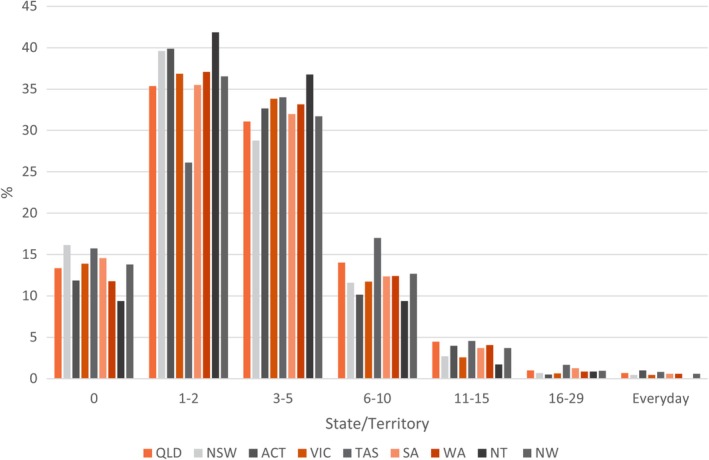
State/territory and nationwide (NW) range of days of pelvic pain per month, over the last 6 months reported by students AFAB. For numerical values please refer to the supplementary data.

Students were asked to report on the average number of days per month that they experienced any pelvic pain or discomfort over the last 6 months (Figure 2). Nationwide, 49.7% of students experience an average of 3 or more days of pelvic pain each month, and 18.0% of students experience six or more days. Tasmanian students reported the highest rate of 3 or more days of pain each month (58.1%), followed by students in QLD (51.3%) and WA (51.1%). NSW has the highest prevalence of students with pain on 2 or less days each month (55.7%).

### 4.3 Metropolitan and Regional/Rural Pelvic Pain Disparities

**FIGURE 3:**
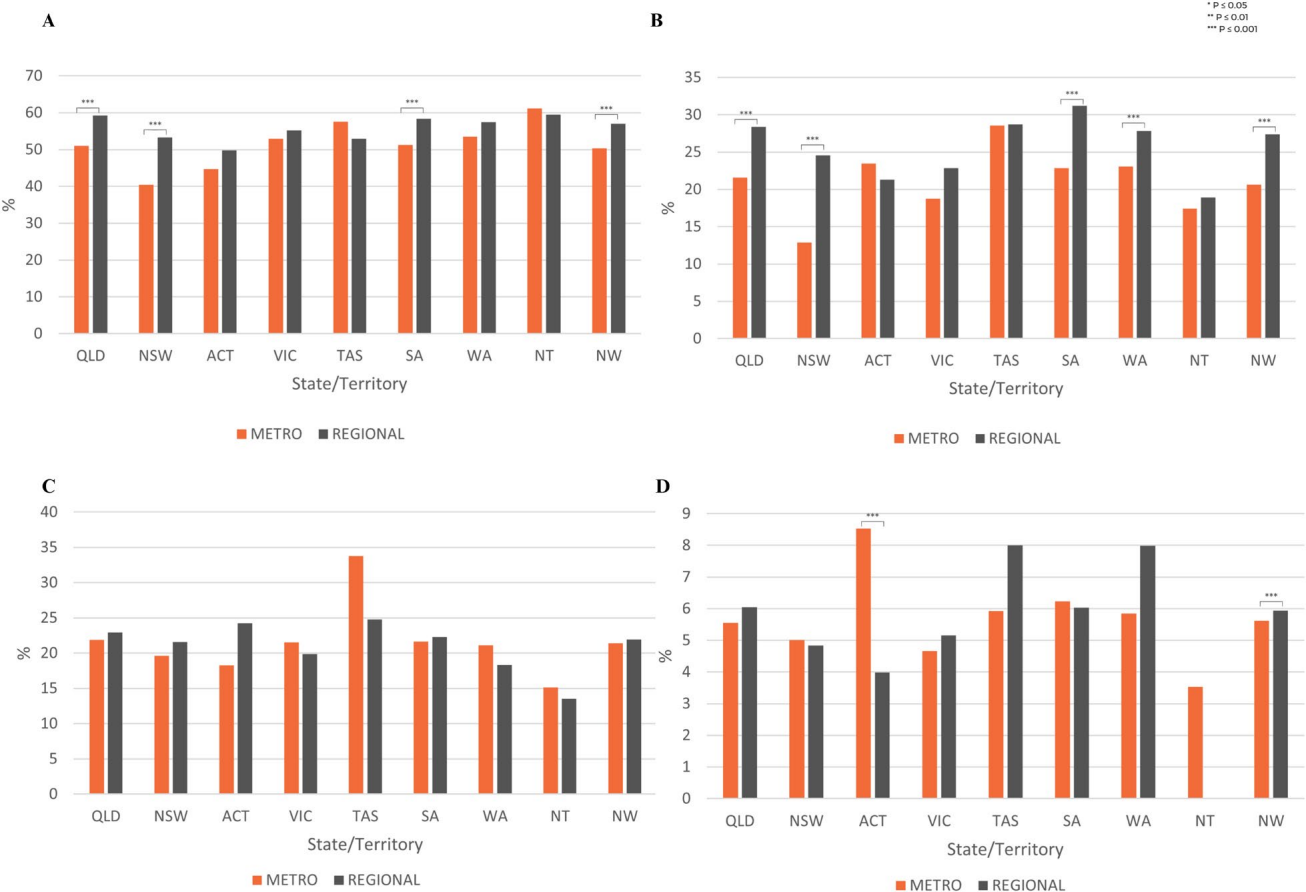
Metropolitan and Non‐Metropolitan State and Territory Comparison. (A) Had regular severe period pain. (B) Regularly missed school or work because of their period. (C) Seen a health professional. (D) Been to an emergency department. Comparison made between metropolitan and non‐metropolitan groups using chi‐squared tests. *, *p* ≤ 0.05, **, *p* ≤ 0.01, ***, *p* ≤ 0.001. For numerical values please refer to the supplementary data.

In QLD, NSW and SA more students in regional areas experienced significantly more regular severe pain (*p* ≤ 0.001) (Figure 3). For all four questions pertaining to period and pelvic pain impact, regional QLD's prevalence was higher than their metropolitan counterparts. The most significant trend was seen in more regional students regularly missing school or work because of their period for all states/territories except the ACT. In TAS (33.8%), WA (21.1%) and the NT (15.12%), more metropolitan students accessed a health professional for their period/pelvic pain. With regional TAS (8.0%) and WA (8.0%) students; and metropolitan students in the ACT (8.5%) having the highest prevalence of ED presentations.

### 4.4 Prevalence of Pelvic Pain by School Type

**FIGURE 4:**
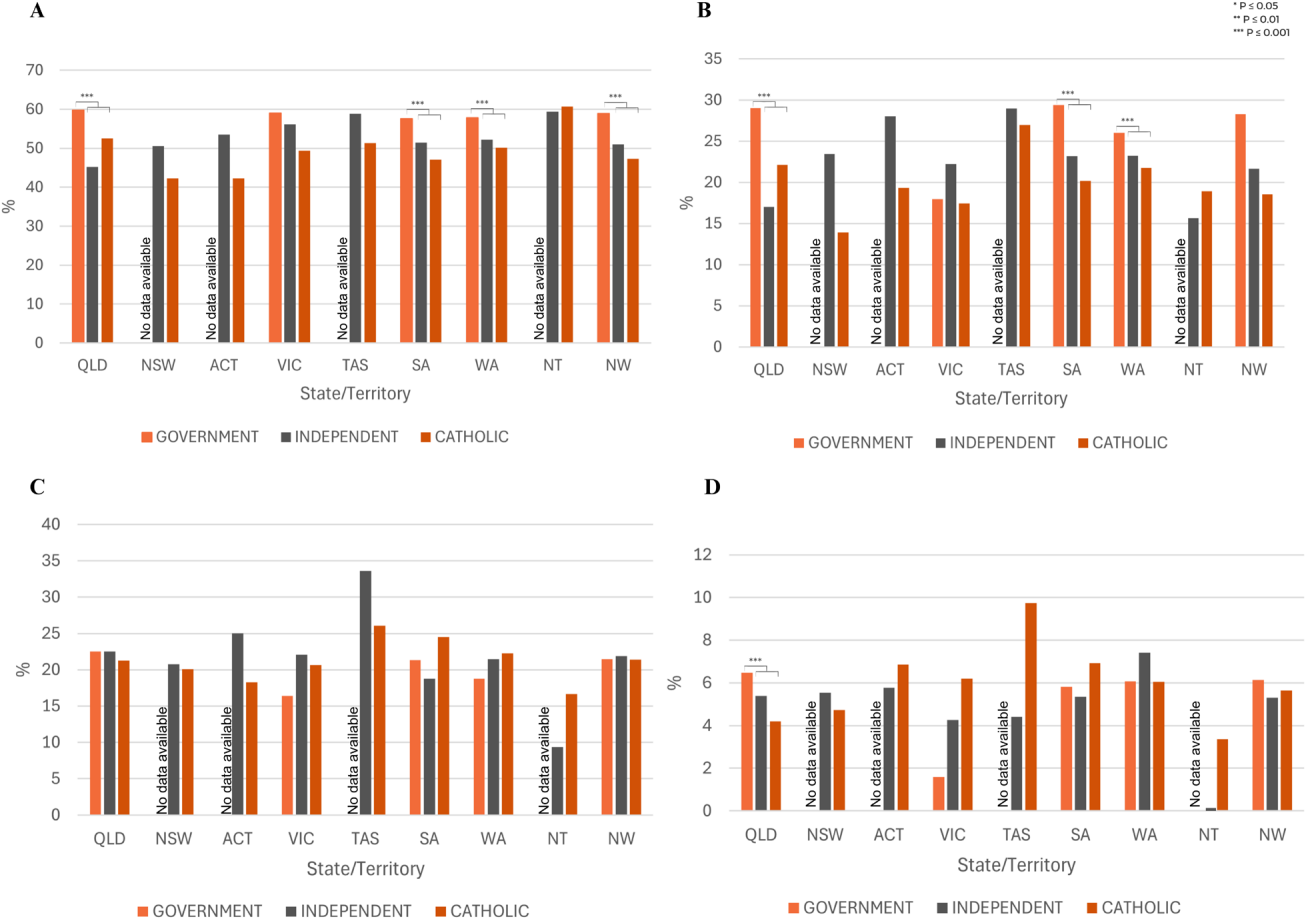
School Type State and Territory. (A) Had regular severe pain. (B) Regularly missed school or work because of their period. (C) Seen a health professional. (D) Been to an emergency department. Comparison was made between catholic and independent, and government schools using chi‐squared tests. ‘No data available’ is shown in states/territories that did not provide Government schools with funding for PPEP Talk. *, *p* ≤ 0.05, **, *p* ≤ 0.01, ***, *p* ≤ 0.001. For numerical values please refer to the Supporting Information.

Pelvic and period pain impact was assessed across Government, Independent and Catholic schooling systems. Figure 4A demonstrates a clear trend, that, where public school data was available more students at Government schools experienced regular severe period pain across all States. As a national average 58.72% of Government, 50.99% of Independent, 47.30% of Catholic school students experience regular severe period pain (*p* ≤ 0.001). All States except Victoria had higher levels of school absenteeism with periods in Government schools compared to independent and catholic schools (*p* ≤ 0.001). QLD, SA and WA received the most support from respective State Governments, giving greater sample sizes, greater power and stronger statistical significance. All three states demonstrated a significantly greater prevalence of students from Government schools experiencing regular severe period pain and school absenteeism due to periods, compared to Catholic and Independent schools (*p* ≤ 0.001).

Presentations to HP or ED for period or pelvic pain varied between school type and state/territory. The highest prevalence of students presenting to a health professional and ED came from Tamanian Independent schools (33.6%) and Catholic schools (9.7%) respectively. Independent schools in the Northern Territory had the lowest rates of presentation to HPs (9.4%) and ED (0%).

### 4.5 ICSEA Quartile and Pelvic Pain Disparities

**FIGURE 5:**
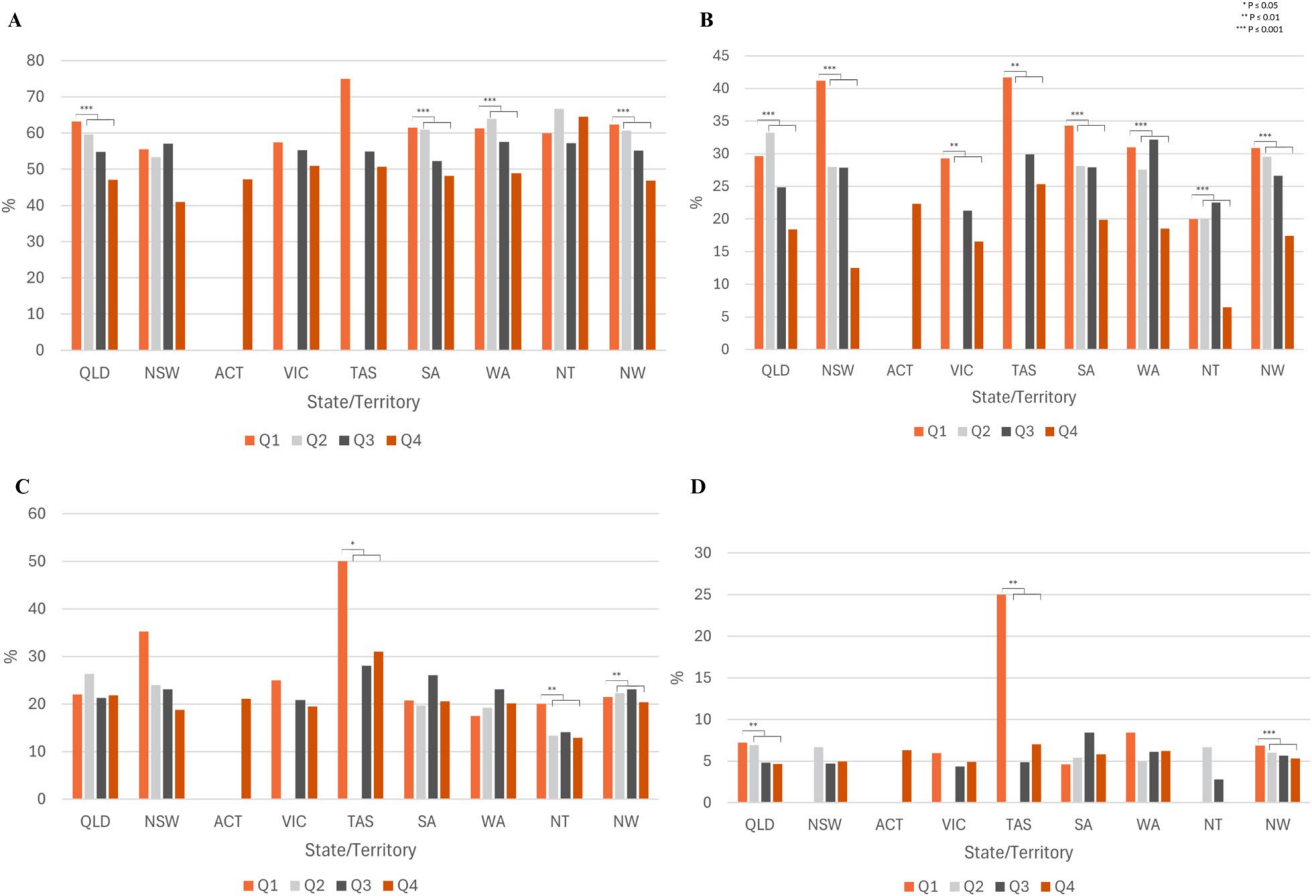
Period and Pelvic Pain by ICSEA Quartile. Q1–Q4 represent schools in the lowest to highest quartiles of socioeconomic status. (A) Had regular severe pain. (B) Regularly missed school or work because of their period. (C) Seen a health professional. (D) Been to an emergency department. The lowest ICSEA quartile (Q1) was compared to the upper three quartiles (Q2, Q3 and Q4) using chi‐squared tests. *, *p* ≤ 0.05, **, *p* ≤ 0.01, ***, *p* ≤ 0.001. For numerical values please refer to the Supporting Information.

ICSEA (Index of Community Socio‐educational Advantage) values were assigned to each school by the Australian Curriculum, Assessment and Reporting Authority (ACARA). Values were assigned from July 2022–June 2023 data and split according to ACARA quartiles. Responses to the four key questions were compared between the lowest quartile (Q1) and the upper three quartiles (Q2, Q3 and Q4) (Figure 5). In ACT only schools in the highest quartile were seen, due to funding, and Victoria had no data from schools in the second quartile. As a national average, all four questions pertaining to period/pelvic pain were highest for schools in the lowest ICSEA quartile. For all States and Territories students in low ICSEA schools experienced high rates of school absenteeism due to their period when compared to high ICSEA schools. The students from low ICSEA schools in TAS had the highest impact of period and pelvic pain across all four questions. In the lowest quartile ICSEA schools in Tasmania, 75% of students reported experiencing regular, severe period pain; 42% regularly missed school or work because of their period; 50% had presented to a health professional for period or pelvic pain and 25% had presented to ED.

### 4.6 PPEP Talk Feedback

As a national average, over 99% of students AFAB reported that the PPEP Talk was informative and showed them tools to use if they get period pain. More than 97% of students said that the PPEP Talk taught them things they didn't know about the human body and had information they could use to improve their general health. Nationally the proportion of students who knew what endometriosis was rose from 47.8% pre PPEP Talk to 95.5% post PPEP Talk. In the financial year 2022–2023, 1233 school staff completed and consented to sharing the responses from their evaluation. 100% of schools who received the PPEP Talk want it to return.

## 5 Discussion

### 5.1 National Impact of Australia's Largest Schools‐Based Pain Program

PPEP Talk has shown that over half (52.67%) of Australian adolescents AFAB experience regular severe period pain. Over one in five students reported regularly missing school or work because of their period (22.96%). Published research has shown that every day counts regarding future economic and educational achievement [10]. Declines in academic achievement, school engagement, and an increase in social isolation are associated with any level of absence [10]. In addition, school‐based presenteeism, where a child attends school but is unwell, also results in negative impacts on education, mental and physical health [11].

The PPEP Talk provides strategies for self‐management of pain, increased awareness of the potential that endometriosis may be a contributing factor and provides information on symptoms that should be discussed with a health practitioner. By building self‐efficacy and confidence in pain management strategies, PPEP Talk aims to decrease absenteeism and increase presenteeism in school students and into the workforce.

### 5.2 Dysmenorrhoea as a Predictor of Future Persistent Pain Conditions

Severe dysmenorrhoea among adolescents is associated with an increased prevalence of persistent pain conditions in later years [12, 13]. These conditions include both PPP and widespread pain conditions including fibromyalgia. Effective management of dysmenorrhea in early years reduces this risk [14].

More than half of students in TAS, QLD and WA reported experiencing three or more days of pelvic pain or discomfort each month for at least the last 6 months. 5.3% of adolescents nationwide reported experiencing pain on 10 or more days per month, already fulfilling the diagnostic criteria for PPP. PPEP Talk offers the opportunity to recognise students at risk of developing persistent pain conditions and target interventions at a pivotal time of development to maximise the likelihood of living a life with less pain. Fundamentally, pain education can lead to reports of lower pain intensity and higher expectations of recovery [15, 16]. Investment in primary and secondary prevention leads to reduced healthcare spending and improved outcomes [17].

### 5.3 Compounded Socioeconomic Disadvantage Across Australian Demographics

PPEP Talk® has identified concerning disparities in period and pelvic pain across Australian communities. Predictive demographic factors were metropolitan, non‐metropolitan, independent, catholic and government schools and ICSEA values.

While dysmenorrhea and pelvic pain are common across all Australian schools, disadvantaged regional, Government and low ICSEA school students bear an increased burden when compared with metropolitan, private and high ICSEA schools. When comparing students from low and high ICSEA value schools, 59% versus 49% reported experiencing regular severe period pain and 28% versus 20% regularly miss school or work because of their period. The pain experienced by these students compounds their socioeconomic disadvantage. The lack of financial support provided by the NSW, ACT and NT state governments unfortunately led to reduced services to their public‐school students with pain. This is despite information that across Australia public school students suffer a higher rate of dysmenorrhea and pelvic pain than private school equivalents. Students at public schools are 20% more likely to experience regular severe period pain compared to private schools (increasing from 49% to 59%).

### 5.4 Presentations to Health Professionals and Emergency Departments

Greater prevalence of period and pelvic pain did not result in greater engagement with health professionals. 21.6% of students had engaged with a health professional and 5.7% had presented to ED for period/pelvic pain. Due to health literacy and access to services students from low ICSEA schools, having reported more pain, have lower presentations to HP and higher presentations to ED compared to students from a higher ICSEA school [18]. For TAS and NT students in the lowest socioeconomic group, more regular severe pain and school absenteeism coincided with greater presentation to a HP and ED. By providing a first explanation of the neuroscience underlying dysmenorrhea, endometriosis, and pelvic pain, PPEP Talk may facilitate help‐seeking behaviour and future discussions with health practitioners. This allows directed and higher‐level discussions to streamline a student's interactions with health services through knowledge and pain education.

## 6 Strengths and Limitations

To our knowledge, this is the largest data cohort describing the prevalence of period and pelvic pain among adolescents. A major strength is the lack of bias or sampling error. Data was collected prior to receiving the PPEP Talk presentation and recorded each students' subjective experience of pain. The study collated cross‐sectional data from all students at schools, whether or not they experienced period/pelvic pain. A limitation of this study is the subjective nature of pain and self‐reporting. As a schools‐based program, we were unable to determine which students had or will develop endometriosis over their lifetime.

In summary, PPEP Talk has identified that period and pelvic pain are common among Australian students. Nationally 52.7% of students reported experiencing regular severe period pain. 23.0% of students reported regularly missing school or work with their period. 5.3% of students reported experiencing 10 or more days of pelvic pain each month, fulfilling the criteria for PPP. The impact of period and pelvic pain is greater in non‐metropolitan, Government, and low socioeconomic schools. PPEP Talk must be accessible for all schools to reduce health inequity rather than propagating it with inequitable funding across states/territories. The Pelvic Pain Foundation of Australia was commissioned by National Action Plan for Endometriosis to deliver PPEP Talk to Australian students to address NAPE Priority 1 (Education and Awareness). This study outlines our findings as a baseline to guide future allocation of education and services for Australian students.

## Conflicts of Interest

Kate Tomsett and Susan Evans receive financial remuneration from the Pelvic Pain Foundation of Australia. There are no additional conflicts of interest.

## Supporting information


**Data S1.** Supplementary Information.

**Data S2.** Supplementary Information.

## Data Availability

The data that supports the findings of this study are available in the Supporting Information material of this article.
