# Supplementary material for: Prevalence and Impact of Period and Pelvic Pain in Australian Adolescents: The PPEP Talk Schools Program
Source: Aust N Z J Obstet Gynaecol. 2025 Mar 14;66(1):e70015. doi: 10.1111/ajo.70015 (PMC12867549; doi:10.1111/ajo.70015)
Supplement: Supplementary file 1 — Data S1. Supplementary Information. [file AJO-66-0-s001.pdf]

Pre-PPEP Talk evaluation for people assigned female at birth (Qld)

Instructions: please only colour in one box for each question, do not tick or cross

School:

Age:

○ 12 or younger ○ 13 ○ 14 ○ 15 ○ 16 ○ 17 ○ 18 or older

What gender do you identify as?

○ Female ○ Male ○ Transgender ○ Non-binary Not Listed: ______

Do you identify as:

○ Aboriginal ○ Torres Strait Islander ○ Both ○ Neither

1. During your life, have you had at least one period?

○ Yes ○ No

Over the last 6 months, have you:

2. Had regular severe period pain?

○ Yes ○ No

3. Had significant interference to your daily activities because of your period?

○ Yes ○ No

4. Experienced bowel or bladder pain?

○ Yes ○ No

5. Regularly missed school or work because of your period?

○ Yes ○ No

6. Felt sure there is something wrong with your periods?

○ Yes ○ No

7. How many days on average a month would you have pelvic pain or discomfort of any kind? (out of 30 days)

○ 0 ○ 1-2 ○ 3-5 ○ 6-10 ○ 11-15 ○ 16-29 ○ Everyday

Over your lifetime have you:

8. Seen a health professional (GP, physiotherapist, psychologist, dietitian etc.) about period/pelvic pain?

○ Yes ○ No

9. How many times have you seen a health professional about period/pelvic pain?

○ 0 ○ 1 ○ 2 - 5 ○ 6 - 10 ○ more than 10

10. Been to an emergency department at a hospital for period/pelvic pain?

○ Yes ○ No

11. How many times have you been to an emergency department for pelvic pain?

○ 0 ○ 1 ○ 2 - 5 ○ 6 - 10 ○ 10 or more

12. Do you know what endometriosis is?

○ Yes ○ No ○ I don't know

PREVIEW

Post-PPEP Talk evaluation for people assigned female at birth - Please complete at the end of the session

Now that you've listened to the PPEP Talk...

13. Do you know what endometriosis is?

◯ Yes ◯ No ◯ I don't know

14. Are you likely to visit a health professional about your period/pelvic pain?

◯ Yes ◯ No ◯ Don't have pain

Your opinion of the PPEP Talk Program

16. PPEP Talk was informative

◯ Yes ◯ No ◯ Somewhat

17. PPEP Talk taught me things about the human body that I didn't know

◯ Yes ◯ No ◯ Somewhat

18. PPEP Talk showed me tools to use if I get period pain

◯ Yes ◯ No ◯ Somewhat ◯ Don't have pain

19. PPEP Talk had information on how to improve my general health

◯ Yes ◯ No ◯ Somewhat

If you have any comments or suggestions about PPEP Talk please add them below

If you would like more information about PPEP Talk Next Steps, please add your email here

Do you consent to this information being used anonymously for research purposes?

◯ Yes ◯ No

Visit us at Pelvic Pain Foundation of Australia - www.pelvicpain.org.au

PREVIEW
