# Supplementary material for: Prevalence and Impact of Period and Pelvic Pain in Australian Adolescents: The PPEP Talk Schools Program
Source: Aust N Z J Obstet Gynaecol. 2025 Mar 14;66(1):e70015. doi: 10.1111/ajo.70015 (PMC12867549; doi:10.1111/ajo.70015)
Supplement: Supplementary file 2 — Data S2. Supplementary Information. [file AJO-66-0-s002.pdf]

Figure 2

How many days on average a month would you have pelvic pain or discomfort of any kind? (out of 30 days)	0	1 - 2	3 - 5	6 - 10	11 - 15	16 - 29	Everyday
**QLD n (%)**	519 (13.37)	1372 (35.35)	1207 (31.10)	545 (14.04)	174 (4.48)	38 (0.98)	26 (0.67)
**NSW n (%)**	321 (16.15)	787 (39.59)	572 (28.77)	231 (11.62)	54 (2.72)	14 (0.70)	9 (0.45)
**ACT n (%)**	48 (11.88)	161 (39.85)	132 (32.67)	41 (10.15)	16 (3.96)	2 (0.50)	4 (0.99)
**VIC n (%)**	212 (13.90)	562 (36.85)	516 (33.84)	179 (11.74)	39 (2.56)	10 (0.66)	7 (0.46)
**TAS n (%)**	38 (15.77)	63 (26.14)	82 (34.02)	41 (17.01)	11 (4.56)	4 (1.66)	2 (0.83)
**SA n (%)**	367 (14.56)	895 (35.52)	806 (31.98)	312 (12.38)	93 (3.69)	32 (1.27)	15 (0.60)
**WA n (%)**	240 (11.78)	756 (37.10)	676 (33.17)	253 (12.41)	83 (4.07)	18 (0.88)	12 (0.59)
**NT n (%)**	11 (9.40)	49 (41.88)	43 (36.75)	11 (9.40)	2 (1.71)	1 (0.85)	0 (0.00)
**NW n (%)**	1756 (13.81)	4645 (36.53)	4034 (31.73)	1613 (12.69)	472 (3.71)	119 (0.94)	75 (0.59)

Figure 3

	**Had regular severe period pain?**			**Regularly missed school or work because of your period?**			**Seen a health professional (GP, physiotherapist, psychologist, dietitian etc.) about period/pelvic pain?**			**Have been to an emergency department at a hospital for period/pelvic pain?**		
	**METRO**	**REGIONAL**	**p value**	**METRO**	**REGIONAL**	**p value**	**METRO**	**REGIONAL**	**p value**	**METRO**	**REGIONAL**	**p value**
**QLD n (%)**	1089 (51.01)	1061 (59.21)	**p ≤ 0.001**	461 (21.56)	510 (28.41)	**p ≤ 0.001**	468 (21.88)	412 (22.94)	p > 0.05	118 (5.56)	108 (6.05)	p > 0.05
**NSW n (%)**	520 (40.40)	393 (53.32)	**p ≤ 0.001**	167 (12.90)	181 (24.56)	**p ≤ 0.001**	253(19.64)	160 (21.56)	p > 0.05	64 (5.00)	36 (4.84)	p > 0.05
**ACT n (%)**	93 (44.71)	100 (49.75)	p > 0.05	49 (23.44)	43 (21.29)	p > 0.05	38 (18.27)	49 (24.26)	p > 0.05	18 (8.53)	8 (3.98)	p ≤ 0.05
**VIC n (%)**	605 (52.98)	228 (55.21)	p > 0.05	215 (18.74)	94 (22.87)	p > 0.05	247 (21.53)	82 (19.85)	p > 0.05	53 (4.67)	21 (5.16)	p > 0.05
**TAS n (%)**	88 (57.52)	53 (53.00)	p > 0.05	44 (28.57)	29 (28.71)	p > 0.05	52 (33.77)	25 (24.75)	p > 0.05	9 (5.92)	8 (8.00)	p > 0.05
**SA n (%)**	966 (51.22)	406 (58.33)	**p ≤ 0.001**	431 (22.86)	218 (31.19)	**p ≤ 0.001**	408 (21.63)	156 (22.29)	p > 0.05	117 (6.23)	42 (6.03)	p > 0.05
**WA n (%)**	833 (53.47)	292 (57.48)	p > 0.05	361 (23.08)	140 (27.83)	**p ≤ 0.001**	330 (21.09)	93 (18.34)	p > 0.05	91 (5.84)	40 (7.98)	p > 0.05
**NT n (%)**	52 (61.18)	22 (59.46)	p > 0.05	15 (17.44)	7 (18.92)	p > 0.05	13 (15.12)	5 (13.51)	p > 0.05	3 (3.53)	0 (0.00)	p > 0.05
**NW n (%)**	4246 (50.37)	2555 (57.03)	**p ≤ 0.001**	1743 (20.64)	1222 (27.38)	**p ≤ 0.001**	1809 (21.42)	982 (21.94)	p > 0.05	473 (5.62)	263 (5.94)	**p ≤ 0.001**

Figure 4

	Had regular severe period pain?				Regularly missed school or work because of your period?				Seen a health professional (GP, physiotherapist, psychologist, dietitian etc.) about period/pelvic pain?				Have been to an emergency department at a hospital for period/pelvic pain?			
	**GOV**	**INDEP**	**CATH**	**p value***	**GOV**	**INDEP**	**CATH**	**p value***	**GOV**	**INDEP**	**CATH**	**p value***	**GOV**	**INDEP**	**CATH**	**p value***
**QLD n (%)**	1333 (59.75)	339 (45.16)	462 (52.56)	**p ≤ 0.001**	648 (29.05)	143 (16.99)	173 (22.14)	**p ≤ 0.001**	501 (22.54)	138 (22.54)	231 (21.26)	p > 0.05	143 (6.47)	27 (4.19)	55 (5.40)	p > 0.05
**NSW n (%)**	NA	584 (50.56)	317 (42.29)		NA	193 (23.45)	148 (13.91)		NA	278 (20.76)	131 (20.09)		NA	65 (4.73)	35 (5.53)	
**ACT n (%)**	NA	104 (53.55)	83 (42.28)		NA	48 (28.03)	44 (19.35)		NA	45 (25.00)	39 (18.29)		NA	17 (6.86)	9 (5.77)	
**VIC n (%)**	75 (59.06)	320 (56.08)	424 (49.38)	p > 0.05	23 (17.97)	113 (22.21)	169 (17.44)	p > 0.05	21 (16.41)	135 (22.09)	167 (20.64)	p > 0.05	2 (1.60)	40 (6.19)	32 (4.27)	p > 0.05
**TAS n (%)**	NA	59 (58.82)	80 (51.30)		NA	31 (28.99)	40 (26.96)		NA	30 (33.58)	46 (26.09)		NA	11 (9.74)	6 (4.41)	
**SA n (%)**	79 (57.63)	375 (51.42)	289 (47.05)	**p ≤ 0.001**	354 (29.38)	161 (23.19)	131 (20.18)	**p ≤ 0.001**	258 (21.34)	195 (18.79)	106 (24.50)	p > 0.05	70 (5.82)	55 (6.93)	30 (5.34)	p > 0.05
**WA n (%)**	695 (57.75)	345 (52.21)	177 (50.15)	**p ≤ 0.001**	268 (26.02)	150 (23.24)	79 (21.74)	**p ≤ 0.001**	193 (18.77)	155 (21.47)	73 (22.24)	p > 0.05	62 (6.07)	42 (6.06)	25 (7.42)	p > 0.05
**NT n (%)**	NA	54 (59.38)	19 (60.67)		NA	17 (15.63)	5 (18.89)		NA	15 (9.38)	3 (16.67)		NA	3 (3.37)	0 (0.00)	
**NW n (%)**	2778 (58.84)	2180 (50.99)	1851 (47.30)	**p ≤ 0.001**	1334 (28.26)	856 (21.66)	789 (18.52)	**p ≤ 0.001**	1011 (21.45)	991 (21.86)	796 (21.40)	p > 0.05	287 (6.13)	260 (5.56)	192 (5.30)	p > 0.05

*p values found using a Chi squared test to compare Government School data to combined Independent and Catholic School data

NA – data was not available due to funding and permissions

Figure 5

	**Had regular severe period pain?**					**Regularly missed school or work because of your period?**					**Seen a health professional (GP, physiotherapist, psychologist, dietitian etc.) about period/pelvic pain?**					**Have been to an emergency department at a hospital for period/pelvic pain?**				
	**Q1**	**Q2**	**Q3**	**Q4**	**p value***	**Q1**	**Q2**	**Q3**	**Q4**	**p value***	**Q1**	**Q2**	**Q3**	**Q4**	**p value***	**Q1**	**Q2**	**Q3**	**Q4**	**p value***
**QLD n(%)**	800 (63.19)	281 (59.66)	259 (54.76)	786 (47.07)	**$p \leq 0.001$**	377 (29.64)	158 (33.19)	117 (24.84)	307 (18.41)	**$p \leq 0.001$**	280 (22.03)	125 (26.32)	101 (21.26)	366 (21.84)	$p > 0.05$	92 (7.21)	33 (6.90)	23 (4.82)	78 (4.65)	**$p \leq 0.01$**
**NSW n(%)**	10 (55.56)	40 (53.33)	251 (57.05)	601 (40.94)	$p > 0.05$	7 (41.18)	21 (28.00)	122 (27.85)	185 (12.52)	**$p \leq 0.001$**	6 (35.29)	18 (24.00)	102 (23.08)	278 (18.78)	$p > 0.05$	0 (0.00)	5 (6.67)	21 (4.73)	74 (4.98)	$p > 0.05$
**ACT n(%)**	0 (0.00)	0 (0.00)	0 (0.00)	192 (47.29)		0 (0.00)	0 (0.00)	0 (0.00)	91 (22.30)		0 (0.00)	0 (0.00)	0 (0.00)	86 (21.08)		0 (0.00)	0 (0.00)	0 (0.00)	26 (6.31)	
**VIC n(%)**	46 (57.50)	0 (0.00)	265 (55.32)	409 (50.93)	$p > 0.05$	24 (29.27)	0 (0.00)	102 (21.29)	133 (16.56)	**$p \leq 0.01$**	21 (25.00)	0 (0.00)	100 (20.83)	157 (19.45)	$p > 0.05$	5 (5.95)	0 (0.00)	21 (4.37)	40 (4.92)	$p > 0.05$
**TAS n(%)**	9 (75.00)	0 (0.00)	89 (54.94)	36 (80.70)	$p > 0.05$	5 (41.67)	0 (0.00)	49 (29.88)	18 (25.35)	**$p \leq 0.01$**	6 (50.00)	0 (0.00)	46 (28.05)	22 (30.99)	**$p \leq 0.05$**	3 (25.00)	0 (0.00)	8 (4.85)	5 (7.04)	**$p \leq 0.01$**
**SA n(%)**	253 (61.56)	212 (60.92)	313 (52.25)	579 (48.25)	**$p \leq 0.001$**	140 (34.31)	98 (28.08)	168 (27.91)	239 (19.90)	**$p \leq 0.001$**	85 (20.73)	69 (19.66)	156 (26.04)	247 (20.53)	$p > 0.05$	19 (4.59)	19 (5.40)	51 (8.44)	70 (5.80)	$p > 0.05$
**WA n(%)**	195 (61.32)	181 (63.92)	195 (57.25)	539 (48.96)	**$p \leq 0.001$**	98 (31.01)	78 (27.56)	110 (32.16)	204 (18.51)	**$p \leq 0.001$**	56 (17.55)	54 (19.22)	79 (23.10)	223 (20.14)	$p > 0.05$	27 (4.59)	14 (4.95)	21 (6.12)	69 (6.21)	$p > 0.05$
**NT n(%)**	3 (60.00)	10 (66.67)	40 (57.14)	20 (64.52)	$p > 0.05$	1 (20.00)	3 (20.00)	16 (22.54)	2 (6.45)	**$p \leq 0.001$**	1 (20.00)	2 (13.33)	10 (14.08)	4 (12.90)	$p > 0.05$	0 (0.00)	1 (6.67)	2 (2.82)	0 (0.00)	$p > 0.05$
**NW n(%)**	1316 (62.37)	724 (60.66)	1412 (55.11)	3162 (46.84)	**$p \leq 0.001$**	652 (30.87)	358 (29.50)	684 (26.65)	1179 (17.44)	**$p \leq 0.001$**	455 (21.48)	268 (22.30)	594 (23.09)	1383 (20.40)	**$p \leq 0.01$**	146 (6.86)	72 (6.00)	147 (5.69)	362 (5.32)	**$p \leq 0.001$**

* p values found using a Chi squared test to compare Q1 vs Q2, Q3 and Q4
